# Apolipoprotein E gene polymorphism: effects on plasma lipids and risk of type 2 diabetes and coronary artery disease

**DOI:** 10.1186/1475-2840-11-36

**Published:** 2012-04-23

**Authors:** Rajesh Chaudhary, Atip Likidlilid, Thavatchai Peerapatdit, Damras Tresukosol, Sorachai Srisuma, Suphachai Ratanamaneechat, Charn Sriratanasathavorn

**Affiliations:** 1 Department of Biochemistry, Faculty of Medicine Siriraj Hospital, Mahidol University, Bangkok 10700, Thailand; 2 Department of Medicine, Faculty of Medicine Siriraj Hospital, Mahidol University, Bangkok 10700, Thailand; 3 Department of Physiology, Faculty of Medicine Siriraj Hospital, Mahidol University, Bangkok 10700, Thailand; 4 Department of Preventive and Social Medicine, Faculty of Medicine Siriraj Hospital, Mahidol University, Bangkok 10700, Thailand; 5 Her Majesty's Cardiac Center, Faculty of Medicine Siriraj Hospital, Mahidol University, Bangkok 10700, Thailand

**Keywords:** Apolipoprotein E, Polymorphism, Type 2 diabetes mellitus, Hyperglycemia, Coronary artery disease, Restriction fragment length polymorphism

## Abstract

**Background:**

The most common apolipoprotein E (apoE) gene polymorphism has been found to influence plasma lipid concentration and its correlation with coronary artery disease (CAD) has been extensively investigated in the last decade. It is, however, unclear whether apoE gene polymorphism is also associated with increased risk of type 2 diabetes mellitus (T2DM). The knowledge of this study may provide the primary prevention for T2DM and CAD development before its initiation and progression. Therefore, this study was carried out to determine the association between apoE gene polymorphism and T2DM with and without CAD and its role in lipid metabolism.

**Methods:**

The case-control study was carried out on a total of 451 samples including 149 normal control subjects, 155 subjects with T2DM, and 147 subjects with T2DM complicated with CAD. The apoE gene polymorphism was tested by polymerase chain reaction-restriction fragment length polymorphism (PCR-RFLP). Univariable and multivariable logistic regression analyses were used to identify the possible risks of T2DM and CAD.

**Results:**

A significantly increased frequency of E3/E4 genotype was observed only in T2DM with CAD group (p = 0.0004), whereas the ε4 allele was significantly higher in both T2DM (p = 0.047) and T2DM with CAD (p = 0.009) as compared with controls. E3/E4 genotype was also the independent risk in developing CAD after adjusting with established risk factors with adjusted odds ratio (OR) 2.52 (95%CI 1.28-4.97, p = 0.008). The independent predictor of individuals carrying ε4 allele still remained significantly associated with both CAD (adjusted OR 2.32, 95%CI 1.17-4.61, p = 0.016) and T2DM (adjusted OR 2.04, 95%CI 1.07-3.86, p = 0.029). After simultaneously examining the joint association of E3/E4 genotype combined with either obesity or smoking the risk increased to approximately 5-fold in T2DM (adjusted OR 4.93, 95%CI 1.74-13.98, p = 0.003) and 10-fold in CAD (adjusted OR 10.48, 95%CI 3.56-30.79, p < 0.0001). The association between apoE genotypes on plasma lipid levels was compared between E3/E3 as a reference and E4-bearing genotypes. E4-bearing genotypes showed lower HDL-C and higher VLDL-C and TG, whereas other values of plasma lipid concentrations showed no significant difference.

**Conclusions:**

These results indicate that ε4 allele has influence on lipid profiles and is associated with the development of both T2DM with and without CAD, and furthermore, it increased the risk among the subjects with obesity and/or smoking, the conditions associated with high oxidative stress.

## Introduction

Type 2 diabetes mellitus (T2DM) is one of the most common diseases with a high incidence and prevalence throughout the world. It affects nearly 4% of the world's population and this percentage will supposedly be increasing up to 5.4% by year 2025 [1]. Prevalence of diabetes in Thai adults as shown by the previous study on Thai population was 9.6% (2.4 million population) and the impaired fasting glucose was 5.4% (1.4 million people). Mean fasting plasma glucose level by age, sex, and area of residence was found to be substantially higher in urban population group than rural [2]. T2DM is also known as a major independent risk factor for coronary artery disease (CAD) and is the major cause of morbidity and mortality affecting people with diabetes. To date, several mechanisms such as dyslipoproteinemia, obesity, oxidative stress, smoking, exercise, alcohol intake, and genetic factors have been identified as risk factors of both T2DM and CAD. Lack of apolipoprotein E (apoE) gene has been clearly demonstrated as a leading cause of severe hyperlipidemia and spontaneous development of atherosclerosis in mammals [3,4]. However, few studies have been able to demonstrate an association between T2DM and various single nucleotide polymorphisms (SNPs) [5]. Recently, Zeljko et al. 2011 [6] indicated that apoE gene polymorphism is also associated with obesity in normal Croatian Roma population. Adipocytes in an obese person which are the central and causal components in T2DM can generate high amount of biologically active molecules called adipokines or adipocytokines such as plasminogen activator inhibitor-1 (PAI-1), resistin, leptin, interleukin-6 (IL-6), and tumor necrosis factor alpha (TNF-α) [7]. These inflammatory cytokines inhibit insulin-stimulated glucose metabolism in skeletal muscles and stimulate gluconeogenesis in hepatocytes causing hyperglycemia [8]. Hyperglycemia-induced oxidative stress results in reducing glucose uptake from blood by muscle cells and develops into insulin resistance by decreasing insulin secretion from pancreatic β-cells [9]. Taken these together, increased oxidative stress in hyperglycemia and reduced lipid clearance because of apoE gene polymorphism are effective in developing insulin resistance and T2DM. In addition, high oxidative stress can also cause vascular inflammation leading to atherosclerosis through several cytokines such as NF-κB, TNF-α, IL-1β, and other proinflammatory cytokines [10]. All of these factors are one of the underlying causes of metabolic syndrome [7]. Grundy reported that subjects suffering from the metabolic syndrome are at 2-fold higher risk of developing CAD and at 5-fold higher risk of developing T2DM; however, persons suffering from T2DM are at 3-fold higher risk of developing CAD [11].

ApoE gene is one of the most studied genes which is responsible for stabilizing and solubilizing circulating lipoproteins in our body and also responsible for the development of CAD [10,11]. ApoE acts as a high affinity ligand for several hepatic lipoprotein receptors such as low-density lipoprotein receptor (LDLR) and LDL-related protein (LRP) and is involved in the process of cellular incorporation of several lipoproteins for transport and digestion [12]. ApoE is a plasma glycoprotein of 34 kDa with 299-amino acids associated with several other plasma glycoproteins, such as high density lipoprotein (HDL), very low density lipoprotein (VLDL), and chylomicrons [13]. In humans, apoE gene located on the chromosome at position 19q13.2 has been known to be polymorphic. SNPs at positions 112 (rs 429358) and 158 (rs 7412) determine three major alleles: ε2 (T to C substitution at position 158), the most common ε3, and ε4 (C to T substitution at position 112); 3 isoforms: ApoE2 (Cys112, 158Cys), ApoE3 (Cys112, 158Arg), and ApoE4 (Arg112, 158Arg); and 6 genotypes having 3 homozygous: E2/E2, E3/E3, E4/E4, and 3 heterozygous: E2/E3, E2/E4, E3/E4 [13]. Previous studies have shown that apoE alleles have influence on the lipid clearance and metabolism in humans. ApoE ε2 allele has been reported to be associated with higher plasma levels of apoE, decreased plasma levels of LDL cholesterol (LDL-C) and lower risk of CAD [14] while apoE ε4 is associated with lower plasma level of apoE, increased plasma levels of total cholesterol (TC), LDL-C, VLDL cholesterol (VLDL-C), and greater risk of CAD when compared to apoE3 homozygotes [15]. One reason for this impaired clearance by apoE ε4 leading to pathogenesis of CAD might be that apoE ε4 binds strongly to LDLR compared to other genotypes. The resulting high amount of lipid can suppress the synthesis of LDLR leading to lower clearance of lipoprotein from our body through LDLR [15]. Other studies have also supported that apoE ε4 allele is associated with the risk of CAD [16]. However, according to a recent meta-analysis, the cardiovascular role of apoE2 is uncertain [17] because of its tendency to increase triglyceride (TG) level [18]. In addition, apo ε2 homozygote in combination with certain additional disorders may develop type III familial hyperlipidemia and premature atherosclerosis [12]. Consequently, with their ability to affect lipid levels, the apoE gene polymorphism could be one of the factors influencing development of both T2DM and CAD. The prevalence of T2DM and CAD is increasing in Thai population according to the cross-sectional ECG survey of 1991 in Thai population, which found that the age-standardized prevalence rate of CAD was 9.9/1000 subjects (men 9.2/1000, women 10.7/1000) [19]. World Health Organization (WHO) global prevalence of diabetes report estimated that by the year 2030, 366 million people, particularly in developing countries, will be affected by diabetes [20]. Genetic factors like apoE are also considered to be genetic determinants of plasma lipoprotein levels and play a central role in the development of CAD. However, it is unclear whether apoE gene is associated with T2DM. The primary aim of this study was to demonstrate the influence of the apoE gene polymorphism on plasma lipids is notable and is an important determinant of T2DM and CAD. Secondarily, this genetic study might also add more information about a Thai population beyond traditional risk factors.

## Materials and methods

### Subjects

The studied subjects were recruited from Siriraj Hospital in Bangkok province. Full consent forms were signed by the subjects after the nature and motif of the study was clearly explained to them. The study protocol was approved by the Ethics Committee of Clinical Study in Humans, Faculty of Medicine Siriraj Hospital, Mahidol University. Questionnaires were used to collect the information of family and medical history, alcohol consumption, smoking habits and physical activity. Other clinical and biochemical data such as dyslipidemia, systolic blood pressure (SBP) and diastolic blood pressure (DBP) from all subjects were obtained from clinical and laboratory examinations. Anthropometric data (weight, height) were collected and used for BMI calculation. Obesity was defined as BMI ≥ 25 kg/m^2 ^according to WHO suggested criteria for Asian populations [21]. Dyslipidemic or hyperlipidemic were defined as when one has level of TC >200 mg/dL, TG >150 mg/dL, LDL-C >130 mg/dL, HDL-C <40 mg/dL, TC/HDL-C ratio >4.0 or under medication of lipid lowering drugs. Cigarette smokers were allowed into the study if they had once smoked even if they were no longer smokers. Alcohol drinkers were defined as those who drank at least two times a week for more than a year. Physical activities were defined as exercise for at least 2 to 3 days/week for at least 30 minutes. Hypertension was defined as blood pressure above 140/90 mmHg or taking antihypertensive drugs. These subjects were categorized into three groups: normal healthy controls, T2DM with and T2DM without CAD, according to the criteria of American Diabetes Association Classification 2010 [22], with an age range from 40-65 years.

The normal healthy control group consisted of 149 subjects with fasting plasma glucose (FPG) < 100 mg/dL from among those who were randomly selected after routine health check-up to screen out those having hyperlipidemia, hypertension, history of chest pain, family history of CAD or any forms of cardiovascular disease, diabetes mellitus, hepatic and renal diseases, inflammation, general illness, traumatic injury, endocrine disease, and other metabolic disorders. Subjects under medication or drug abusers were also excluded.

T2DM subjects without CAD included 155 subjects. All subjects fulfilled the diabetes mellitus diagnostic criteria of FPG≥126 mg/dL or were under treatment with oral antidiabetic drugs. The subjects had no electrocardiogram (ECG) and/or angiography abnormalities, no documented history of CAD and no sign of myocardial ischemia during exercise. Other exclusion criteria for this group were possession of type 1 diabetes mellitus, renal disease, hepatic disease, endocrine disease, and other metabolic diseases.

T2DM complicated with CAD (T2DM + CAD) subjects included 147 subjects. Subjects were confirmed CAD by coronary angiography, with at least 50% stenosis in a major coronary artery or one of their branches. Subjects were diagnosed to have diabetes with FPG≥126 mg/dL or those with drug-treated T2DM. Exclusion criteria included those having renal disease, hepatic disease, type 1 diabetes mellitus, any form of endocrine disease or metabolic disease.

### APOE Genotyping

Blood samples were collected in EDTA containing tubes. Guanidine-HCl precipitation method was performed for genomic DNA extraction. Genomic DNA was subjected to polymerase chain reaction (PCR) with primers specific to apoE gene, sense: 5' AACAACTGACCCCGGTGGCG 3', antisense: 5' ATGGCGCTGAGGCCGCGCTC 3', sense: 5' CCCACCTGCGCAAGCTGCGC 3', using thermal cycler with thermal profile according to Richard et al [23]. In brief, PCR reaction mixture included 20 pmol of each primer, 0.3 μg genomic DNA, 10 mM of each dNTP, 10X PCR buffer, 10% DMSO in a final volume of 50 μL. 10 μL of PCR products was digested with 0.3 unit of Hha1 enzyme according to the supplier's recommended procedure (Biolabs New England). The resulted fragments were then separated on 8% polyacrylamide gel and stained with ethidium bromide. Bands were compared with 25 bp DNA marker and the different individual genotypes were separated and categorized based on the following band length criteria: E2/E2: 91, 83, 61; E3/E3: 91, 61, 48, 35; E4/E4: 72, 61, 48, 35; E2/E3: 91, 83, 61, 48, 35; E2/E4: 91, 83, 72, 61, 48, 35 and E3/E4: 91, 72, 61, 48, 35.

### Lipid analysis and biochemical determination

Venous blood samples were collected from the patients after 12 hours of overnight fast. Plasma (TC), TG, HDL-C, glucose and glycosylated hemoglobin (HbA1c) were quantified using an automated clinical chemistry analyzer and enzyme-based colorimetric lists supplied by Roche Diagnostics, Germany. LDL-C levels were calculated using Friedewald formula [24]. VLDL-C level was obtained by using the following equation, VLDL-C = (TC - LDL-C - HDL-C) and non-HDL-C level was calculated by subtracting HDL-C value from total cholesterol value as a candidate biometrical equivalent to apoB 100 in diabetes [25].

### Statistical analysis

All statistical analyses were performed with IBM SPSS v1 (IBM corporation, Armonk, New York, US) and Microsoft 2010 based Excel (Microsoft, US). All data were expressed as mean (SEM). One-way analysis of variance (ANOVA) followed by posthoc Bonferroni multiple comparison test was applied to evaluate the mean difference of the data between three groups (control, T2DM without CAD, and T2DM with CAD). Independent samples t-tests were applied to evaluate the mean difference in HbA1c, diabetes duration between T2DM and T2DM with CAD and lipid profiles between groups. Categorical data such as sex, hypertension, history of smoking, history of alcohol drinking, physical activity and dyslipidemia were evaluated by Chi-square tests or Fisher's exact tests. Allele and genotype difference between groups and deviations from Hardy-Weinberg equilibrium were tested by Chi-square tests. The association between diseases and polymorphism was provided by crude or univariable logistic regression analysis with unadjusted odds ratio (OR) and 95% confidence interval (95% CI). The adjusted OR with 95% CI was used to determine the independent risk factor for development of diabetes and CAD by multivariable logistic regression analysis after adjusting for age, sex, BMI, smoking habits, and physical activity. The multivariable logistic regression analysis was also repeated for adjusted OR with 95% CI to determine the risk of apoE polymorphism combined with obesity and smoking (joint association) after adjusting for age, sex, and physical activity. Statistical significance was considered as p < 0.05.

## Results

### The baseline characteristics of the study population

All enrolled subjects were Thais recruited from Bangkok area. The anthropometric and demographic data were summarized along with the clinical and biochemical data as shown in Table 1. The data from normal controls were used to compare with the data from T2DM with and without CAD. A significant age difference was found between control and T2DM with CAD groups (p < 0.0001) which particularly signifies that increase in age could possibly lead to higher chances of developing CAD. There was also significant sex difference between controls and CAD (p < 0.0001) due to the high prevalence of males among CAD patients. Other clinical data such as BMI, systolic blood pressure (SBP) and diastolic blood pressure (DBP) were significantly higher in both groups when compared to controls. CAD patients had higher frequencies of smoking (p = 0.033) and lower levels of physical activity (p = 0.030) as compared to controls and T2DM patients. Lipid profile data demonstrated significantly higher levels of TC, LDL-C, VLDL-C, TG, non-HDL-C and lower level of HDL-C in T2DM and CAD patients when compared to controls. Patients with CAD had diabetes for a longer period (p < 0.0001) and showed no significant difference in HbA1c (p = 0.151) as compared to the patients without CAD. Blood glucose levels were also significantly higher in both groups as compared to the controls (p < 0.0001).

**Table 1:** Anthropometric, demographic and clinical data of T2DM with and without CAD compared to normal healthy controls

Variables	Controls (n = 149)	T2DM (n = 155)	T2DM + CAD (n = 147)
**Age (Years)**	52.01 (0.62)	51.95 (0.53)	57.56 (0.47)**

**Sex (male/female)**	49/100	57/98	95/52**

**BMI (kg/m^2^)**	23.84 (0.27)	26.89 (0.31)*	27.49 (0.36)**

**SBP (mmHg)**	111.54 (1.12)	147.31 (1.76)*	155.46 (1.96)**

**DBP (mmHg)**	73.11 (0.86)	84.50 (1.01)*	92.26 (1.20)**

**Hypertension (%)**	-	73.54	93.19

**Smokers (%)**	3.35	4.51	9.52^†^

**Alcohol consumer (%)**	23.80	25.50	32.70

**Physical activity (%)**	68.70	67.09	56.46^†^

**Diabetes duration (Years)**	-	6.09 (0.37)	9.07 (0.62)

**Glucose (mg/dL)**	89.7 (0.64)	225.9 (6.41)*	192.9 (6.55)**

**HbA1c (%)**	-	8.94 (0.16)	8.60 (0.17)

**Triglyceride (mg/dL)**	98.23 (3.68)	221.1 (10.35)*	204.3 (8.70)**

**TC (mg/dL)**	191.52 (1.80)	237.76 (5.47)*	202.48(4.19)^†^

**LDL-C (mg/dL)**	111.31(1.76)	146.65(4.84)*	122.36(3.93)^†^

**HDL-C (mg/dL)**	60.17 (1.23)	50.42 (1.27)*	44.59 (0.95)**

**VLDL-C (mg/dL)**	19.65 (0.73)	45.86 (2.67)*	40.86 (1.74)**

**Non-HDL-C (mg/dL)**	131.35 (1.95)	182.26 (4.46)*	157.88 (4.09)**

**TC/HDL-C**	3.4 (0.1)	4.9 (0.2)*	4.8 (0.1)**

**Dyslipidemia (%)**	-	85.2	100

Data are presented as mean values (SEM), or numbers (n) and percentage of subjects. *p-value < 0.0001 in comparison between controls and T2DM, **p-value < 0.0001 in comparison between controls and T2DM + CAD, †p-value < 0.05 in comparison between controls and T2DM + CAD. *BMI*: body mass index; *SBP*: systolic blood pressure; *DBP*: diastolic blood pressure; *HbA1c*: hemoglobin A1c; *TC*: total cholesterol; *LDL-C*: low density lipoprotein cholesterol; *TG*: triglyceride; *HDL-C*: high density lipoprotein cholesterol; *VLDL-C*: very low density lipoprotein cholesterol.

### ApoE genotype and allele frequencies

The genotype distribution of both controls and CAD patients were in Hardy-Weinberg equilibrium except for T2DM that deviated from the basic norm (p = 0.0001). This was due to the lower presence of E2/E3 genotype in diabetic group (Table 2). However, since it was unlikely that any of the assumptions for Hardy-Weinberg equilibrium were violated, such a departure was attributed to chance. E3/E3 is also the most common genotype in Thai general population. E3/E3 genotype and ε3 allele were significantly lower (p = 0.003 and 0.010 respectively) while E3/E4 genotype was significantly higher only in T2DM with CAD subjects (p = 0.0004). In T2DM subjects, E3/E4 genotype had a tendency to be higher (19.35% vs. 14.09%) but showed no significant difference (p = 0.219). In addition, E2/E3 genotype and ε2 allele were also significantly lower in T2DM patients (p = 0.005 and 0.010, respectively). However, ε4 allele frequency manifested itself as significantly higher in both T2DM and CAD patients as compared to the controls (p = 0.047 and 0.009, respectively) (Table 3).

**Table 2:** Frequency distribution of apoE genotypes and alleles in Hardy-Weinberg Equilibrium

Genotype	Controls (n = 149)	T2DM (n = 155)	T2DM + CAD (n = 147)
**E2/E2**	2 (1.34%)	1 (0.64%)	1 (0.68%)

**E3/E3**	113 (75.83%)	117 (75.48%)	88 (59.86%)

**E4/E4**	1 (0.67%)	4 (2.58%)	1 (0.68%)

**E2/E3**	12 (8.05%)	2 (1.29%)	11 (7.48%)

**E2/E4**	0 (0%)	1 (0.64%)	0 (0%)

**E3/E4**	21 (14.09%)	30 (19.35%)	46 (31.29%)

**p-value**	0.198	0.0001	0.157

**Allele ε2(95% CI)**	0.05(0.03 - 0.08)	0.02(0.01 - 0.04)	0.04(0.02 - 0.07)

**Allele ε3(95% CI)**	0.87(0.82 - 0.90)	0.86(0.81 - 0.89)	0.79 (0.74 - 0.83)

**Allele ε4(95% CI)**	0.07(0.05 - 0.11)	0.13 (0.09 - 0.16)	0.16 (0.12 - 0.21)

**Table 3:** Genotype and Allele frequencies distribution of apoE gene polymorphism in controls, T2DM with and without CAD compared to healthy controls

Genotype	Groups		
	**Controls (n = 149)**	**T2DM (n = 155)**	**T2DM + CAD (n = 147)**

**E2/E2**	0.013	0.006	0.006

**E3/E3**	0.758	0.754	0.598**

**E4/E4**	0.007	0.025	0.007

**E2/E3**	0.080	0.012*	0.074

**E2/E4**	0	0.006	0

**E3/E4**	0.140	0.193	0.321***

**Allele ε2**	0.05	0.02*	0.04

**Allele ε3**	0.87	0.86	0.79**

**Allele ε4**	0.07	0.13*	0.16^††^

*p-value < 0.01 compared between controls and T2DM, **p-value < 0.01, ***p-value < 0.001 compared between controls and T2DM + CAD.

### Association of apoE gene polymorphism and diseases

Low lipid clearance property of ε4 allele may possibly make it an independent risk factor for the development of CAD and T2DM. Thus, E3/E4 genotype may be the risk factor for development of CAD and/or diabetes. The univariate analysis was used to determine these associations according to the genotype and allele frequencies of apoE gene polymorphism.

Interestingly, E3/E4 genotype increased the risk of CAD with unadjusted OR 2.78 (95%CI 1.50-5.16, p = 0.0004) and showed no association with T2DM with unadjusted OR 1.46 (95%CI 0.76-2.81, p = 0.219). The ε4 allele appeared to increase risk of both T2DM and CAD with unadjusted OR 1.72 (95%CI 0.97-3.06, p = 0.047) and 2.37 (95%CI 1.36-4.15, p = 0.0009, respectively) (Table 4). After being adjusted for age, sex, BMI, smoking habits, and physical activity using multivariable binary logistic regression analysis as shown in Table 5 the E3/E4 genotype appeared to be the independent risk factor for development of CAD with adjusted OR 2.52 (95%CI 1.28-4.97, p = 0.008). However, the E3/E4 genotype was not found to be an independent risk factor for diabetes, but the ε4 allele was the independent risk factor of both T2DM and CAD with adjusted OR 2.04 (95%CI 1.07-3.86, p = 0.029) and OR 2.32 (95%CI 1.17-4.61, p = 0.016), respectively. The multivariable analysis of apoE4 polymorphism and the risk of T2DM and CAD were also determined according to anthropometric and demographic characteristics. Sex (particularly male gender), age and BMI were also independent risks for CAD but only BMI was the independent risk for T2DM (Table 5). From joint association analysis, E3/E4 genotype was found to further increase the risk for development of diabetes and CAD when combined with either obesity or smoking after being adjusted for age, sex, and physical activity. The risk factor for T2DM increased from adjusted OR 1.42 (95%CI 0.72-2.78, p = 0.310) (Table 5) to adjusted OR 4.93 (95%CI 1.74-13.98, p = 0.003) (Table 6), whereas CAD risk increased from adjusted OR 2.52 (95%CI 1.28-4.97, p = 0.008) (Table 5) to adjusted OR 10.48 (95%CI 3.56-30.79, P < 0.0001) (Table 6). However, the number of E3/E4 genotypes combined with both obesity and smoking in T2DM with and without CAD was small; thus the statistical power of this joint association comparison is limited.

**Table 4:** Associations of apoE gene polymorphisms with the risk of T2DM and CAD compared to healthy controls represented as unadjusted OR

Genotype	Controls (n = 149)	T2DM (n = 155)	Unadjusted OR (95% CI)	p-value	T2DM + CAD (n = 147)	Unadjusted OR (95% CI)	p-value
**E2/E2**	2	1	0.48 (0.02 - 6.78)	0.616	1	0.50 (0.02 - 7.16)	1.000

**E3/E3**	113	117	0.98 (0.56 - 1.71)	0.942	88	0.48 (0.28 - 0.81)	0.003

**E4/E4**	1	4	3.92 (0.41 - 93.19)	0.371	1	1.01(0.06 - 16.36)	1.000

**E2/E3**	12	2	0.15 (0.02 - 0.72)	0.005	11	0.92(0.36 - 2.33)	0.854

**E2/E4**	0	1	-	1.000	0	-	-

**E3/E4**	21	30	1.46 (0.76 - 2.81)	0.219	46	2.78(1.50 - 5.16)	0.0004

**Allele ε2**	16	5	0.29 (0.09 - 0.85)	0.011	13	0.81(0.36 - 1.81)	0.580

**Allele ε3**	259	266	0.91 (0.56 - 1.48)	0.691	234	0.57(0.36 - 0.90)	0.107

**Allele ε4**	23	39	1.72 (0.97 - 3.06)	0.047	49	2.37(1.36 - 4.15)	0.0009

**Table 5:** Association of apoE gene polymorphism with the risk of T2DM and CAD compared to healthy controls represented as adjusted OR

	T2DM		T2DM + CAD	
	**Adjusted OR (95% CI)^††^**	**p-value**	**Adjusted OR (95% CI)^††^**	**p-value**

**E3/E4 genotype**	1.42 (0.72 - 2.78)	0.310	2.52 (1.28 - 4.97)	0.008^††^

**ε4 allele**	2.04 (1.07 - 3.86)	0.029^††^	2.32 (1.17 - 4.61)	0.016^††^

**Age**	1.00 (0.97 - 1.04)	0.887	1.13 (1.08 - 1.18)	<0.0001

**Sex**	1.29 (0.77 - 2.14)	0.326	3.10 (1.75 - 5.49)	<0.0001

**BMI**	3.68 (2.25 - 6.00)	<0.0001	3.66 (2.09 - 6.39)	<0.0001

**Smoking**	2.58 (0.87 - 7.66)	0.086	0.59 (0.13 - 2.50)	0.473

**Physical activity**	0.68 (0.40 - 1.19)	0.148	0.67 (0.36 - 1.24)	0.206

^††^After adjusting for age, sex, BMI, smoking habit and physical activity. Normal reference ranges of clinical and biochemical data are described in materials and methods.

**Table 6:** Joint association study to evaluate the risk of T2DM and CAD compared to controls

Genotype	Obesity/Smoking	Controls	T2DM	Adjusted OR (95% CI)^††^	p-value	T2DM + CAD	Adjusted OR (95% CI)^††^	p-value
**E3/E3**	Neither	70	34	1	-	30	1	-

**E3/E3**	Either	40	73	3.65 (2.07 - 6.43)	<0.0001	56	2.24 (1.15 - 4.35)	0.018

**E3/E3**	Both	2	10	10.05 (2.06 - 48.96)	0.004	1	2.02 (0.16 - 25.01)	0.585

**E3/E4**	Neither	14	14	1.97 (0.84 - 4.64)	0.118	7	1.02 (0.34 - 3.06)	0.970

**E3/E4**	Either	6	15	4.93 (1.74 - 13.98)	0.003	36	10.48 (3.56 - 30.79)	<0.0001

**E3/E4**	Both	6	1	-	-	3	-	-

^†† ^After adjusting for age, sex and physical activity. Normal reference ranges of clinical and biochemical data are described in materials and methods.

### Relationship between Lipid profiles and apoE4-bearing genotypes

The significant differences in apo E distribution among these three groups are mainly due to the differences in frequencies of the ε4 allele. These distributions in apo ε4 allele frequencies may lead to the differences in plasma lipid levels. To test this hypothesis, we analyzed the correlation between apoE4 and plasma lipid levels (Table 7). There were no significant differences between E4 carriers (E2/E4, E3/E4, E4/E4) and E3/E3 genotype as a reference for all values of plasma lipid levels in both control and T2DM with CAD. In T2DM group, there were significant elevation in the values of VLDL-C and TG but there were no significant differences in TC, LDL-C, HDL-C and non-HDL-C levels in E4 carriers. After pooling the overall subjects, the results showed that E4 carrier has significantly elevated VLDL-C and TG levels while HDL-C concentration was significantly decreased as compared to E3/E3.

**Table 7:** Fasting lipid concentration according to apoE4-bearing genotypes

	Control		T2DM		T2DM + CAD		Total	
**Variables (mg/dL)**	**E3/E3**	**E4-bearing genotypes**	**E3/E3**	**E4-bearing genotypes**	**E3/E3**	**E4-bearing genotypes**	**E3/E3**	**E4-bearing genotypes**

	(n = 113)	(n = 22)	(n = 117)	(n = 35)	(n = 88)	(n = 47)	(n = 318)	(n = 104)

**TC**	191.58 (2.02)	194.09 (5.22)	232.80 (5.33)	234.77 (8.65)	206.52 (5.26)	192.04 (7.96)	210.88 (2.72)	206.86 (5.11)

**LDL-C**	110.87 (1.97)	118.52 (4.45)	145.14 (4.54)	140.91 (8.43)	125.33 (4.84)	113.80 (7.33)	127.48 (2.39)	123.92 (4.59)

**HDL-C**	60.07 (1.43)	55.77 (2.51)	50.84 (1.13)	48.63 (2.98)	45.49 (1.27)	42.70 (1.37)	52.64 (0.81)	47.46 (1.37)**

**VLDL-C**	20.04 (0.87)	20.18 (1.69)	41.38 (1.99)	55.77 (5.85)*	41.25 (2.37)	38.91 (3.04)	33.76 (1.17)	40.62 (2.72)**

**TG**	100.20 (4.33)	100.91 (8.48)	206.92 (9.99)	278.83 (29.63)*	206.26 (11.78)	194.53 (15.21)	168.82 (5.88)	203.10 (13.62)**

**Non-HDL-C**	131.50(2.16)	138.32(4.99)	181.97(5.36)	186.14(8.12)	161.03(5.24)	149.34(7.44)	158.24 (2.83)	159.39 (4.82)

E4-bearing genotypes = E3/E4, E4/E4, E2/E4. Independent sample t-test test was applied to compare between E3/E3 and E4-bearing genotypes. Data are presented as mean values (SEM).

*p-value < 0.05 in comparison between E3/E3 and E4-bearing genotypes in T2DM.

**p-value < 0.01 in comparison between E3/E3 and E4-bearing genotypes in total subjects.

## Discussion

It has been reported that dyslipidemia or dyslipoproteinemia might strongly contribute towards aggravating the problems of micro- and macroangiopathic complications in diabetic patients [26,27]. This implication was mainly characterized by higher prevalence of male gender, older age, smoking habits, and less physical activity. Other differences were duration of diabetes, lower levels of HDL-C and hypertension when compared to those without CAD which were similar to this study. However, some studies have also shown that elevation of TG and non-esterified fatty acid (NEFA) levels accelerated the pathogenesis of T2DM [28-30]. This suggests that dyslipidemia is associated with both T2DM and CAD. Genetic factors which have strong impact on the metabolism of plasma lipid have been studied regarding the potential effect of T2DM and cardiovascular outcomes in various diabetic and non-diabetic subjects. These studies have underscored that apoE gene encoding apolipoprotein E is associated with significant variation in lipid profile in our body [31]. ApoE gene is one of the most widely studied candidate genes for CAD or any other form of cardiovascular disease and/or diabetes. A significant relationship of apo E polymorphism and CAD has been observed in several ethnic groups, including Caucasian in the USA [32], Austrian [33], Finnish [34], Italian [35], Turkish [36], Indian [37] and Chinese [38] populations. Some studies have shown apoE ε4 allele as an independent risk factor after further adjustment of other established risk factors for development of CAD in T2DM [16] and myocardial infraction [37] patients. However, no independent association was observed after adjusting for age, sex, smoking, BMI, HDL-C and TG in African- Americans and Caucasians [39]. Therefore, it is of great interest to study the independent risk factors of this gene polymorphism in a Thai population. The present study also coincides with a previous study regarding the development of CAD in T2DM subjects indicating that the frequency of ε4 allele is significantly higher in CAD compared to controls and can be one of the factors for the progression of CAD disease [40]. The frequencies of apoE allele and genotype vary between different populations [13]. In this study, ε3 allele was the most frequent allele in control, diabetes, and CAD subjects, while ε2 was less frequent. E3/E3 genotype was the most frequent isoform found in all groups compared to the other genotypes which corresponds to previous reports [13,41]. Additionally, the higher frequency of E3/E4 genotype was observed only in CAD (p = 0.0004), whereas the higher frequency of ε4 allele was observed in both T2DM and CAD (p = 0.047, p = 0.0009, respectively). From this study we can conclude that among these selected apoE alleles (ε2, ε3, and ε4), ε4 allele is one of the predictors of both diseases in Thai subjects. In addition, the PDAY (Pathobiological Determinants of Atherosclerosis in Youth) study reported that individuals with E2/E3 genotype had fewer atherosclerotic lesions, whereas those with the E3/E4 had more lesions in the abdominal aorta [42]. These observations strongly suggest that the ε2 allele has a protective role against atherosclerosis. However, in this study, we found that E2/E3 genotype and ε2 allele were also significantly lower in T2DM group indicating that the protective effect of ε2 allele on the development of hyperlipidemia might be less in T2DM patients. It is probable that other environmental and genetic factors are involved in the pathogenesis of CAD.

This present study is the first report to demonstrate that the ε4 allele containing genotypes is the major predictor of development of both T2DM and CAD. Our study also shows strong association of E3/E4 genotype with development of CAD in T2DM patients (Table 4) as reported in previous studies [16,42]. After adjusting for age, sex, smoking, BMI, and physical activity, E3/E4 containing subjects showed 2.52-fold higher risk (p = 0.008) while ε4 allele containing genotypes led to a 2.32-fold higher risk (p = 0.016) for developing CAD and ε4 allele containing genotypes led to a 2.04-fold higher risk (p = 0.029) in the development of T2DM as compared to the controls. This signifies that other risk factors have no influence in development or curbing the disease, suggesting that ε4 allele is the independent risk factor for both T2DM and CAD. However, E3/E4 genotype further increases the risk for the development of diabetes and CAD when combined with smoking and/or obesity (Table 6). This confirms the findings that progression and development of diabetes and CAD is the consequence of multifactorial parameters, for example, obesity, smoking, oxidative stress, defect in pancreatic β-cells, alcohol consumption, exercise, hypertension and genetic factors. In addition, incidence of T2DM is also subject to gene-environment interactions, for example, dietary factors, intake of vegetable fat, polyunsaturated fatty acid, dietary fiber (particularly cereal fiber), magnesium, and caffeine were significantly inversely correlated and intakes of trans fat, saturated fatty acid and heme-iron, glycemic index, and glycemic load were significantly positively correlated with the incidence of T2DM [43]. Similarly, regular exercise has been shown to reduce weight, BMI, HDL-C and insulin resistance. This indicates that exercise is also associated with decrease risk of T2DM and CAD. Furthermore, apoE gene polymorphism has been shown to modulate the effects of exercise on lipoprotein concentrations in plasma [44]. In this study, smoking and obesity are shown to be the two major contributing factors that can promote the development of T2DM and CAD or both when placed in joint association (Table 6). The reason for this is that smoking and obesity enhance the oxidative stress which results in decreased insulin secretion from pancreatic β-cell and decreased uptake of blood glucose into the muscle cells [9,10]. This evidence was supported by the experimental study on atherosclerosis-susceptible B6 (B6.apoE -/-) and atherosclerosis-resistant BALB (BALB.apoE -/-) mice that showed defects in insulin secretion rather than defects in insulin resistance which explains the mark difference in susceptibility to T2DM [45]. Moreover, oxidative stress also increases vascular inflammation leading to CAD and/or any form of cardiovascular disease with or without combination of T2DM [46].

To demonstrate that apoE gene polymorphism is involved in the lipid clearance process and it has great influence on the lipid level in our body [47,48]. Although previous study has shown that non-HDL-C concentration is similar to or better than LDL-C alone in predicting cardiovascular disease (CVD) incidence [49], in this study, the results showed that the decrease in HDL-C and elevation of TG, VLDL-C and LDL-C levels (Table 1) are also the landmarks of diabetes and CAD development as found in an earlier study [50]. However, the association of apoE ε4 allele and lipid profile is still controversial [51]. The ε4 allele has been shown to be associated with high concentration of serum TC and LDL-C in Chinese population [38]. Some studies also showed a significant relationship between E3/E4 genotype with lower HDL-C and higher LDL-C concentrations in CAD patients [52] and with higher TG levels in T2DM patients [48] as compared to the healthy controls. However, a study in the Tunisian population has suggested that HDL-C concentration and other lipid profiles are not associated with apoE gene polymorphism in the total population studied, but has proven that ε4 allele increased LDL-C in type 2 diabetic men [53]. Association of ε4 allele with higher LDL-C and lower HDL-C levels was only found in type 2 diabetic women in Spanish population [54] indicating that gender affects the effect of apoE gene polymorphism. In this study, there is a mean comparison between E4 carriers and E3/E3 genotype in the T2DM and total population studied which showed a significantly higher VLDL-C, TG and lower HDL-C levels in E4 carriers (Table 7) similar to the report of Knouff et al 1999 [15]. Nevertheless, the differences of lipid profiles were not observed in controls and T2DM with CAD. This may be due to dietary restrictions in the controls and lipid-lowering aggressive treatment in T2DM with CAD. Kolovou et al. 2006 [55] reported that the ε4 allele can increase LDL-C concentrations in the presence of an atherogenic diet, but a lower fat diet can suppress this effect. On the other hand, aerobically trained individuals have high HDL and display enhanced glucose tolerance [56]. A lipid-lowering-drug which raises HDL levels and decrease TG levels delays the onset of T2DM and reduces the development of atherosclerosis [57]. In addition, loss of caspase-1 activity which involves the inflammatory process in human atherosclerotic vessel has shown to reduce atherosclerosis lesion formation in Casp1^-/- ^Apo E^-/- ^mice [58]. Another reason for these variations of lipid profiles might be because of the differences in genetic background and the prevalence of high oxidative stress in the studied population. When considering the basis of gene-gene and gene-environment interactions, as described above, the polymorphism study at the genome level might not provide a real picture for development of diseases in respect to lipid profile. A more robust parallel study at the protein level, or of other candidate genes, may be necessary to evaluate the mechanism of T2DM and CAD development. However, this study will provide a good starting point for the screening of large populations before proceeding to the protein level study of apoE and other candidate genes in various races and, additionally, including pharmacogenomics study in the future.

## Conclusions

This study tentatively supports the fact that variability in apoE gene locus is associated with diabetes with and without CAD complication by influencing the plasma lipid levels that are important risk factors for both T2DM and CAD. No single factor can give a satisfactory explanation regarding development of diabetes and CAD as both diseases are complex diseases. Focusing, therefore, on only one risk factor may be less than optimal in enabling researchers to predict who will develop untoward events in complex diseases like diabetes and CAD. Nonetheless, our study strongly supported that the apoE ε4 allele is an independent risk factor for development of both T2DM and CAD. Moreover, obesity and/or smoking, conditions associated with high oxidative stress, can aggravate the progression of both diseases. Genetic studies can thus provide information that may help to improve the ability to identify individuals, families and populations at increased risk, as well as to improve the clinical management of patients with T2DM and CAD. Such information may be useful in developing public health programs reinforcing primary and secondary prevention for T2DM and CAD. Furthermore, T2DM and CAD patients identified to carry high risk genotype or allele should be treated aggressively to prevent the progression of disease.

## Abbreviations

APOE: Apolipoprotein E; CAD: Coronary artery disease; T2DM: Type 2 diabetes mellitus; PCR-RFLP: Polymerase chain reaction-restriction fragment length polymorphism; HDL-C: High-density lipoprotein cholesterol; VLDL-C: Very low density lipoprotein cholesterol; LDL-C: Low-density lipoprotein cholesterol; TC: Total cholesterol; TG: Triglyceride; non-HDL-C: Non-high-density lipoprotein cholesterol; SNPs: Single nucleotide polymorphisms; NF-κB: Nuclear factor kappa B; LDLR: Low density lipoprotein receptor; T: Thymine; C: Cytosine; ECG: Electrocardiogram; WHO: World health organization; SBP: Systolic blood pressure; DBP: Diastolic blood pressure; BMI: Body mass index; FPG: Fasting plasma glucose; Guanidine-HCL: Guanidine-hydrochloride; APO B-100: Apolipoprotein B-100; HbA1c: Glycated hemoglobin A1c; NEFA: Non-esterified fatty acid; PDAY: Pathobiological determinants of atherosclerosis in youth.

## Competing interests

The authors declare that they have no competing interests.

## Authors' contributions

All authors fulfill the criteria for authorship. RC carried out the SNPs analysis. AL conceived all of the study and coordination. RC and AL conducted statistical analysis and drafted manuscript. TP, DT, SR, and CS provided the samples for SNP analysis. AL, TP, DT, SS, and CS participated in study conception and design, interpretation of data and critical revision of manuscript for important intellectual content. All authors read and approved the final version of the manuscripts.
